# Allyl and Benzyl Modified Aramid Nanofibers as an Enhancement in Polystyrene-Based Composites

**DOI:** 10.3389/fchem.2020.586763

**Published:** 2020-11-09

**Authors:** Guo Peng, Wu Yaoqin, Dong Congcong, Sun Changmei, Qu Rongjun, Ji Chunnuan, Zhang Ying, Wang Ying

**Affiliations:** School of Chemistry and Materials Science, Ludong University, Yantai, China

**Keywords:** aramid nanofibers, allyl and benzyl, morphological appearance, mechanical properties, PS

## Abstract

Aramid nanofibers (ANFs) represent the most promising nanoscale building blocks for high-performance nanocomposites. But their applications are mostly limited to those polymers containing –OH or –NH_2_ groups that can interact with ANFs through hydrogen bonding or others. In this paper, allyl and benzyl modified ANFs were successfully fabricated using a metallization method followed by functionalization with allyl and benzyl bromide. A series of modified aramid nanomaterials (ANMs) with different degrees of modification were prepared and their morphologies studied. The modified ANFs were added to polystyrene (PS) films as reinforcements. The mechanical properties of the resulting composite PS films including Young's modulus, toughness and yield strength were dramatically improved compared to those of pure PS film. These new types of reinforcement additives for non-polar polymer materials are presented in this paper.

## Introduction

Aramid fiber is a synthetic fiber composed of a long-chain polyamide with at least 85% of the amide bond directly connected to the benzene rings (Downing and Newell, 2004). Among them, para-aramid fiber, also known as (poly-p-phenylene terephthamide) or PPTA fiber, is particularly widely used (Koo et al., 2019). Aramid fibers have high modulus and high strength, which makes them an ideal mechanical reinforcement for compositing with various materials (Nie et al., 2017). However, there are a few inherent drawbacks with aramid fibers such as flocculation and uneven distribution in the matrix material and weak interfacial bonding strength with the matrix material as a result of its smooth surface and poor dispersibility. Although the weak interfacial bonding issue is partially addressed by its large aspect ratio and high specific surface area, the other issues limit the use of aramid fibers in composites (Jia et al., 2011).

Ultrafine fibers such as nanofibers have higher aspect ratio, higher specific surface area and overall better mechanical properties than macroscopic fibers (Persano et al., 2013). Nanoscale aramid fiber or ANFs combines the advantages of high-performance aramid fiber and nanofiber, therefore its composite effect in the composite materials is greatly enhanced. For example, the addition of aramid fiber to styrene butadiene rubber can effectively inhibit the fatigue crack growth (Yin et al., 2020) or improve mechanical properties of the resulting polymer composite (Gu et al., 2018; Tang et al., 2018).

On the other hand, the high-performance properties of the PPTA fiber, such as high strength, high modulus, high temperature and corrosion resistance, make the synthesis of nanoscale fibers difficult (Yang et al., 2019). In a recent development of the preparation methods, macroscopic aramid fibers were successfully protonated and dispersed in dimethyl sulfoxide (DMSO) to prepare ANFs (Yang et al., 2011). ANFs prepared using this method were subsequently added into matrix materials such as polyurethane (PU) (Kuang et al., 2015; Chen et al., 2016), poly (vinyl alcohol) (PVA) (Guan et al., 2017), polylactic acid (PLA) (Bettini et al., 2017), and polyethylene (PE) (Cai et al., 2019) for performance enhancement.

To further improve the adhesion strength of the ANFs to the matrix material and consequently the mechanical performance of the ANFs, functional groups were introduced on its rigid molecular chain in a variety of ways (Wu and Cheng, 2010). The most common approach is surface chemical grafting, in which the functional group is reacted with the amine group on the aromatic ring or the amide bond (Liu et al., 2010; Dong et al., 2019; Nasser et al., 2019). For example, the mechanical properties of aramid fiber were shown to improve with the introduction of amino groups on its surface (Benrashid and Tesoro, 1990) or chlorosulfonic acid group on the benzene (Lin et al., 2000) ring.

In this paper, benzyl and allyl groups were grafted on the surface of the ANFs to improve the surface wettability, decrease the polarity, and improve the dispersibility in the composite. The morphologies of the synthesized modified ANFs were analyzed, and then a variety of comprehensive analyses were carried out. The modified ANFs were added to polystyrene as reinforcement and the mechanical properties of the resulting composite were evaluated.

## Experimental

### Materials

Bulk Kevlar 964 C was provided by DuPont. DMSO was purchased from Kishida Chemicals (Tokyo, Japan). DMSO was dried with calcium hydride and distilled prior to use. PS with polymerization degree of 104 was obtained from Aladdin Chemical Co. Ltd. KOH was purchased from Aladdin Chemical Reagent Co. Ltd., (ACRC) and were used without further purification. Allyl bromide and benzyl bromide were obtained from Aladdin Chemical Reagent Co. Ltd., and were used as received. The purities of the reagents ranged between 90 and 99%. Water used in this study was deionized water.

### Instrumentation

Elemental analysis was performed on an Elementar Vario EL III element analyzer for the determination of carbon, nitrogen and hydrogen contents. FTIR spectra were collected in the wavenumber range of 700–4,000 cm^−1^ with 64 scans at a 2 cm^−1^ resolution. The microstructures of the R-ANMs were studied by transmission electron microscopy (TEM, Hitachi H-800, Japan). Nitrogen was used as the purge gas. The XRD patterns of samples were collected on a Shimadzu LabX XRD-6100 diffractometer using the Cu K-α radiation. Surface elemental analysis was performed by X-ray photoelectron spectroscopy (XPS) with a Physical Electronics PHI-5702 probe. Each sample was analyzed using Casa XPS software for peak fitting and integration. Thermogravimetric analysis (TGA) was performed on a PerkinElmer TGA 2050 instrument under nitrogen. The heating rate for the polymer composite samples was 10–30 K min^−1^. Tensile measurements of the R-ANMs/PS films were taken with an Instron 4,465 instrument equipped with a 5 KN load cell under the ambient conditions. Each sample was tested at a crosshead speed of 10 mm/min. All samples were cut into strips with an effective gauge length, width and thickness of 30, 10, and 0.26 mm, respectively. Each composite film sample can be divided into five test strips of the same size and thickness, and then the five test strips are measured. The reported values were averaged from three of the five test strips.

### Preparation of ANFs/DMSO Dispersion

ANFs/DMSO dispersion was prepared by splitting the bulk Kevlar 964C threads in DMSO with the aid of KOH as previously reported (Yang et al., 2011).

**Figure 1:**
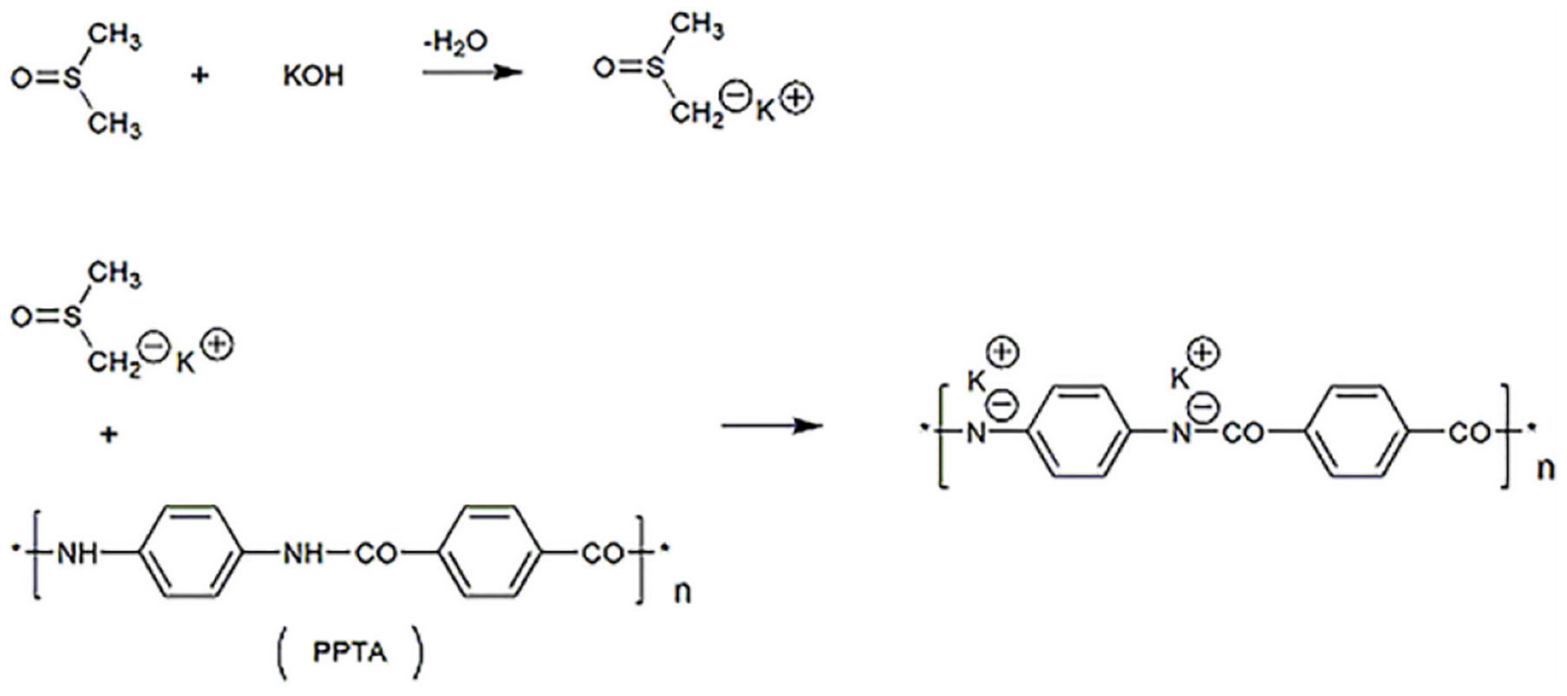
Synthetic route of ANFs/DMSO dispersion.

Typically, 0.6 g of bulk Kevlar 964C threads and 0.9 g of KOH were added into 300 mL of DMSO. The mixture was stirred at room temperature for 1 week and a dark red ANFs/DMSO dispersion was obtained. The metalation reaction is presented in Figure 1.

### Synthesis of R-ANMs

ANMs-C_3_H_5_-X samples with different degrees of allyl and benzyl substitutions were prepared according to the methods described by Takayanagi and Katayose (1981).

The synthetic formulations and characterization results are presented in Table 1.

**Table 1:** Synthetic formulation, element composition and degree of substitution of allyl and benzyl substituted ANFs.

**Sample notation**	**Substitution reagent**	**ANFs volume (mL)**	**Substitution reagent (μL)**	**Elemental composition (%)**			**Degree of substitution (%)**
				**N**	**C**	**O**	
ANMs-C_3_H_5_-1	Allyl bromide	50	145	9.09	61.05	5.189	27.85
ANMs-C_3_H_5_-2		50	291	8.86	61.11	5.503	34.89
ANMs-C_3_H_5_-3		50	727	8.90	67.68	5.506	62.40
ANMs-C_3_H_5_-4		50	1,450	8.74	71.46	5.549	84.63
ANMs-C_3_H_5_-5		50	2,910	8.40	70.06	5.603	91.02
ANMs-C_7_H_8_-1	Benzyl bromide	50	19.99	9.62	66.42	4.879	15.07
ANMs-C_7_H_8_-2		50	39.98	8.48	63.05	5.093	23.92
ANMs-C_7_H_8_-3		50	99.95	8.31	71.42	5.914	43.24
ANMs-C_7_H_8_-4		50	199.90	7.68	72.43	7.417	66.71
ANMs-C_7_H_8_-5		50	399.81	9.01	66.35	5.746	79.57

The degree of substitution in the table is calculated according to the following formula (Kong et al., 2014):

$$\begin{array}{l} DS_{C}=\left[7\left(W_{C}/6\left(W_{N}\right)-7\right)\right]/n \end{array}$$

Where, *DS*_*C*_ is the degree of substitution of R-ANMs calculated by the content of C and N elements; W_*C*_ and W_*N*_ are the mass fraction of C and N elements; n is the number of carbon atoms.

The synthetic procedure for ANMs-C_3_H_5_-1 is described below as an example. In a 100 mL flask, 145 μL of allyl bromide was added into 50 mL of ANFs/DMSO dispersion under nitrogen protection. The reaction was allowed to proceed at 30°C for 16 h with constant stirring. After the reaction, the product polymer was precipitated by pouring into excess water. The precipitate was filtered and washed with water to neutral pH, followed by sequential rinsing with acetone and ethanol. The resulting sediment was dried in a vacuum oven for 5 h.

### Fabrication of R-ANMs/PS Nanocomposite Films

**Figure 2:**
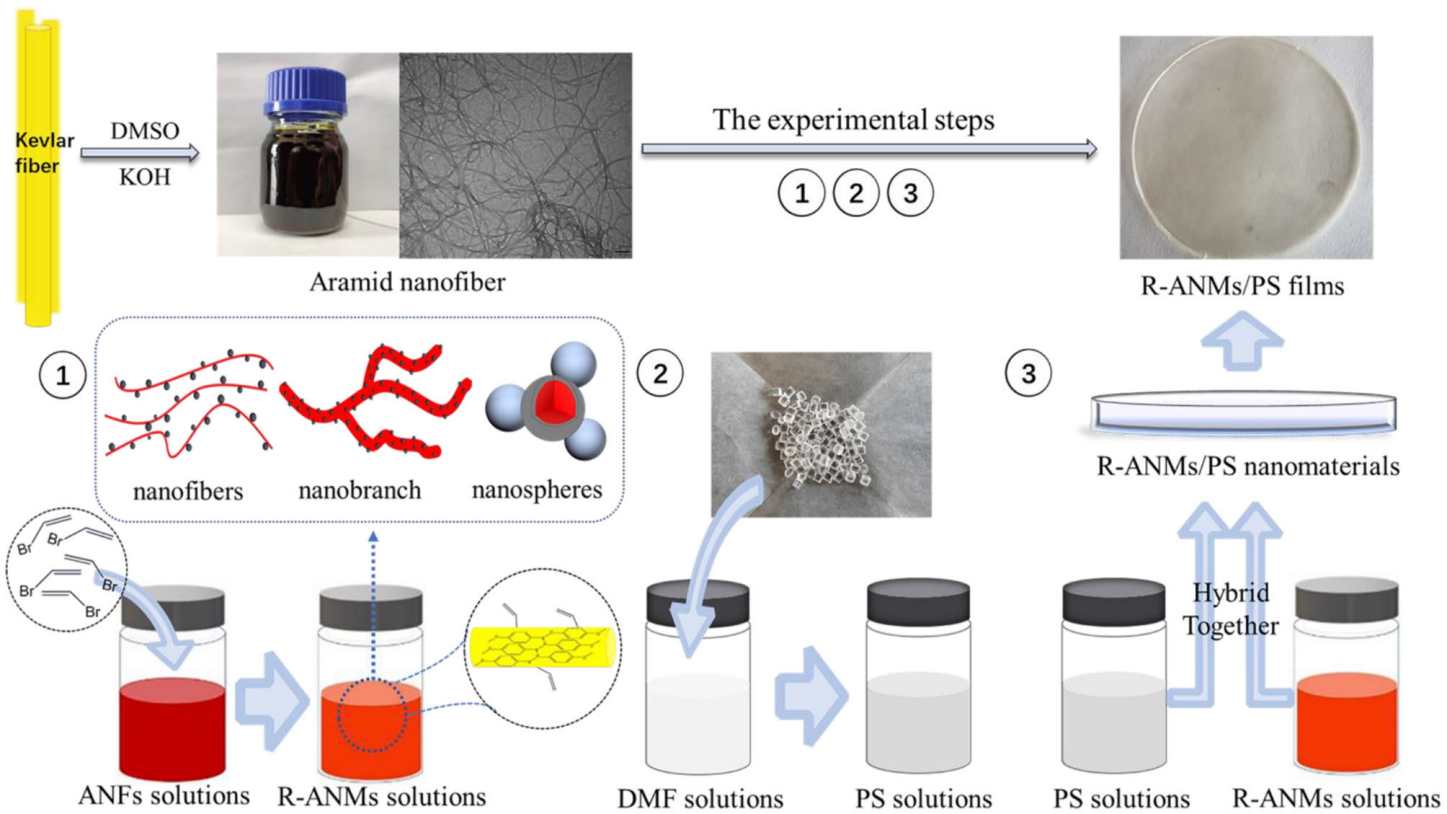
Workflow for fabricating R-ANMs/PS nanocomposite films.

R-ANMs/PS nanocomposite films were prepared by a simple solution casting method. The general workflow is shown in Figure 2. First, 2.0 g of polystyrene was added to 20 mL DMF and stirred for 1.5 h at 50°C to obtain a PS solution with a mass fraction of 10%. A solution of R-ANMs was each prepared in DMSO at 0.3 mg/mL and added to the above solution under continuous stirring for 10 min. The obtained dispersion was sonicated for 15 min and poured into a round Teflon mold with a diameter of 9 cm. After drying in a vacuum oven at 60°C for 48 h, the R-ANMs/PS nanocomposite films formed. The thicknesses of the films were between 0.2 and 0.3 mm by measurement. The R-ANMs/PS films are denoted as ANMs-C_3_H_5_-X/PS and ANMs-C_7_H_8_-X/PS.

## Results and Discussion

### Preparation of the ANFs/DMSO Dispersion

As stated in the literature, Kevlar 964C can be effectively split into aramid nanofibers by deprotonation action in the solution system of DMSO and KOH (Yang et al., 2011). The proposed mechanism is as follows. The strong base KOH reacts with DMSO to form DMSO anions, which break the hydrogen bond between the amide groups of PPTA. Meanwhile, the hydrogen of the amino group of PPTA is abducted and replaced by the potassium ion. This results in positively charged polymer chains, which extend to form microfibers in the DMSO solution due to electrostatic repulsion.

As shown in Figure 2, the ANFs/DMSO dispersion appears red and can exist uniformly and stably for several months. The electron microscopic image of the above dispersion is also shown in Figure 2. The average diameter of the aramid nanofibers is about 25 nm, which is within the range of 20–30 nm stated in the literature.

### Characterization of ANMs-C_3_H_5_-X and ANMs-C_7_H_8_-X

#### FTIR Analysis

**Figure 3:**
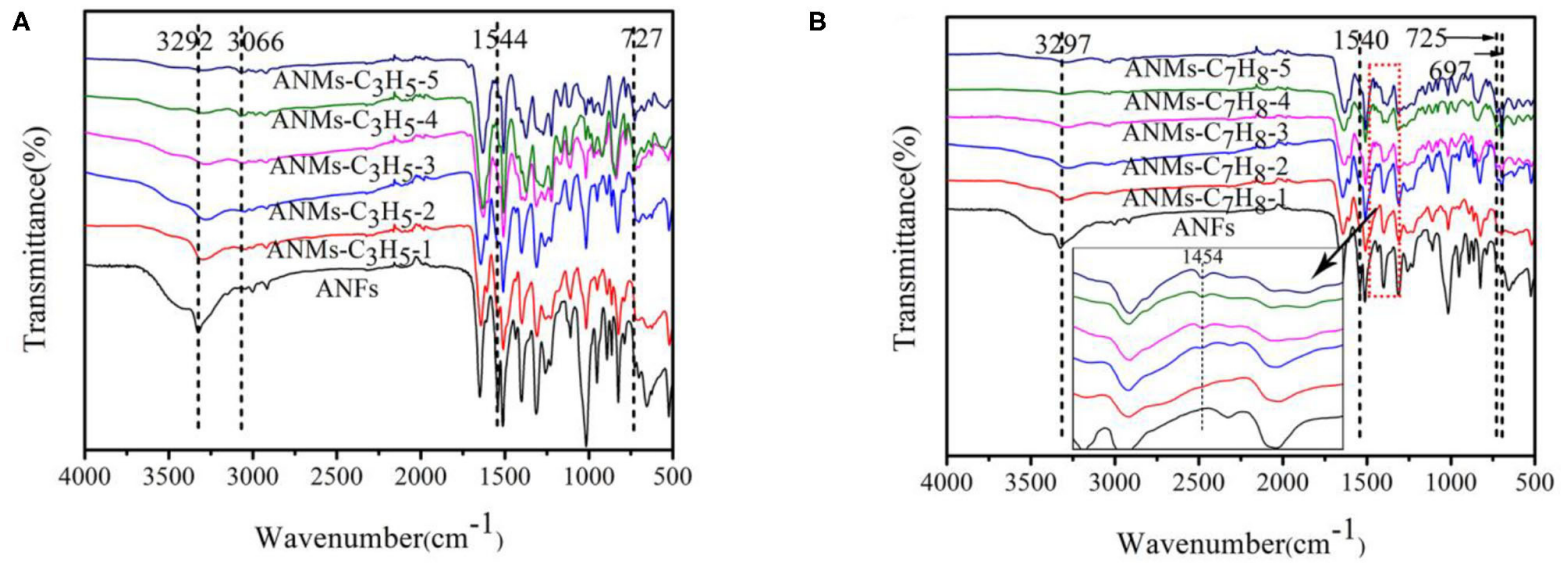
FTIR spectra of ANMs-C_3_H_5_-X **(A)** and ANMs-C_7_H_8_-X **(B)** samples.

The infrared spectra of ANFs, ANMs-C_3_H_5_-X, and ANMs-C_7_H_8_-X are shown in Figure 3, with the spectra of ally substituted ANMs (ANMs-C_3_H_5_-1 to ANMs-C_3_H_5_-5) overlaid in Figure 3A, and benzyl substituted ANMs (ANMs-C_7_H_8_-1 to ANMs-C_7_H_8_-5) in Figure 3B.

With the increase in allyl substitution from ANMs-C_3_H_5_-1 to ANMs-C_3_H_5_-5 in Figure 3A, both the N-H stretching band at 3,292 cm^−1^ and N-H bending band at 1,544 cm^−1^ of the amino groups in ANFs decrease, indicating increasing degrees of deprotonation and destruction of the N-H bond (Changmei et al., 2011; Sun et al., 2011). A similar trend can be observed in the benzyl substituted ANMs in Figure 3B. The ally substituted ANFs see the appearance of characteristic allyl band at 3,066 cm^−1^ as the degree of substitution increases from ANMs-C_3_H_5_-1 to ANMs-C_3_H_5_-5. This suggests that the allyl groups have been successfully grafted to the surface of the ANFs. Similarly, the characteristic bands of aromatic rings at 725 and 697 cm^−1^ appears in the benzyl substituted ANFs with increasing degrees of substitution. As shown in the detailed picture in Figure 3B, the characteristic absorption peak of the benzene-ring skeleton at 1,454 cm^−1^ was observed, and the absorption peak intensity also increased with the increase of benzyl substitution degree (Ebringerová et al., 2015). This proves the successful grafting of the benzyl to the surface of ANFs.

#### XRD Analysis

**Figure 4:**
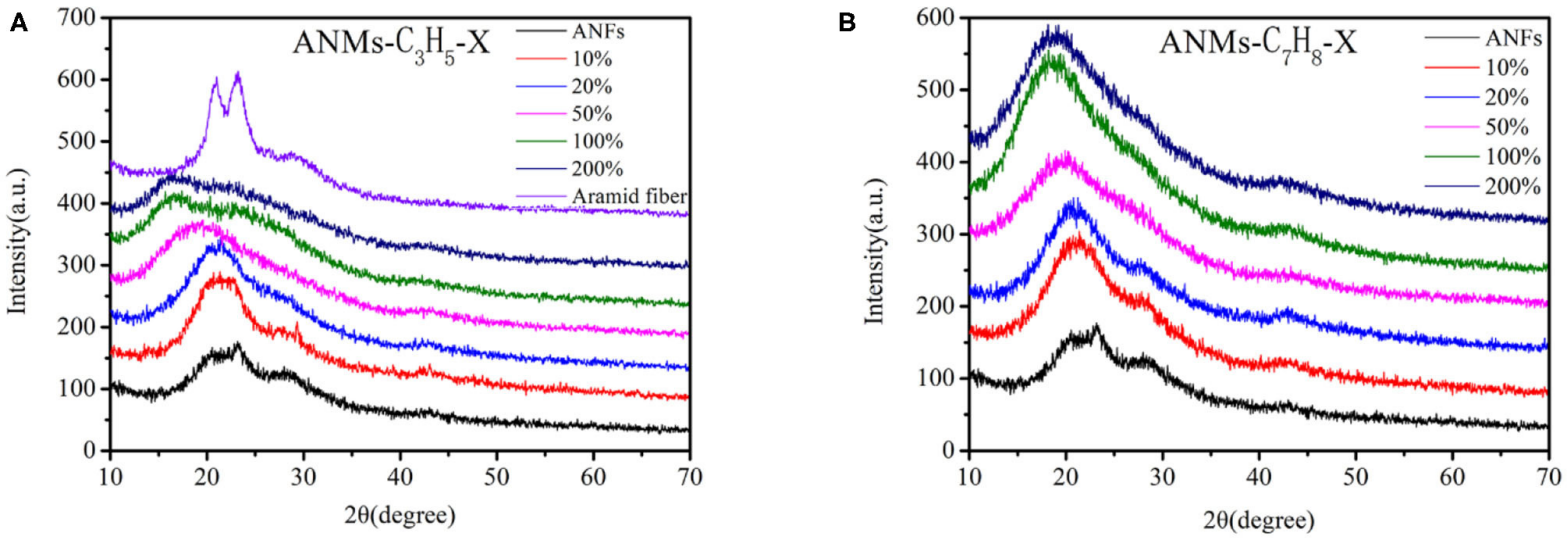
XRD spectra of ANMs-C_3_H_5_-X **(A)** and ANMs-C_7_H_8_-X **(B)** samples.

The wide-angle X-ray diffraction spectra of ANMs-C_3_H_5_-X and ANMs-C_7_H_8_-X at room temperature are shown in Figure 4. The three characteristic peaks of PPTA nanofibers, at 2θ of 19, 23, and 28° correspond to the reflection of planes (110), (200), and (004) (Northolt, 1974), respectively. The axial orientation of PPTA molecule in Kevlar fiber (in Figure 4A) is almost the same as that of PPTA in nanofibers, which indicates that ANFs still retain a large number of crystallization of the macroscopic aramid fibers and maintain their mechanical properties.

With the increase in allyl substitution from ANMs-C_3_H_5_-1 to ANMs-C_3_H_5_-5, the three characteristic peaks broaden and the peak near 20° shifts to a lower angle. When the theoretical grafting rate is >20%, the reflection peak of the (004) surface disappears. These changes indicate the successful substitution of the hydrogen atom of the amide bond by the allyl group (Dong et al., 2018). The introduction of benzyl groups has a considerable effect on the diffraction pattern. With the increase in benzyl substitution from ANMs-C_7_H_8_-1 to ANMs-C_7_H_8_-5, the (004) reflection peak in ANFs almost disappears and one single peak near 20°, which suggests almost amorphous structure, shifts to a lower angle (Ueta et al., 1993). These changes are consistent with the effect of benzyl introduction into ANFs.

#### XPS Analysis

**Figure 5:**
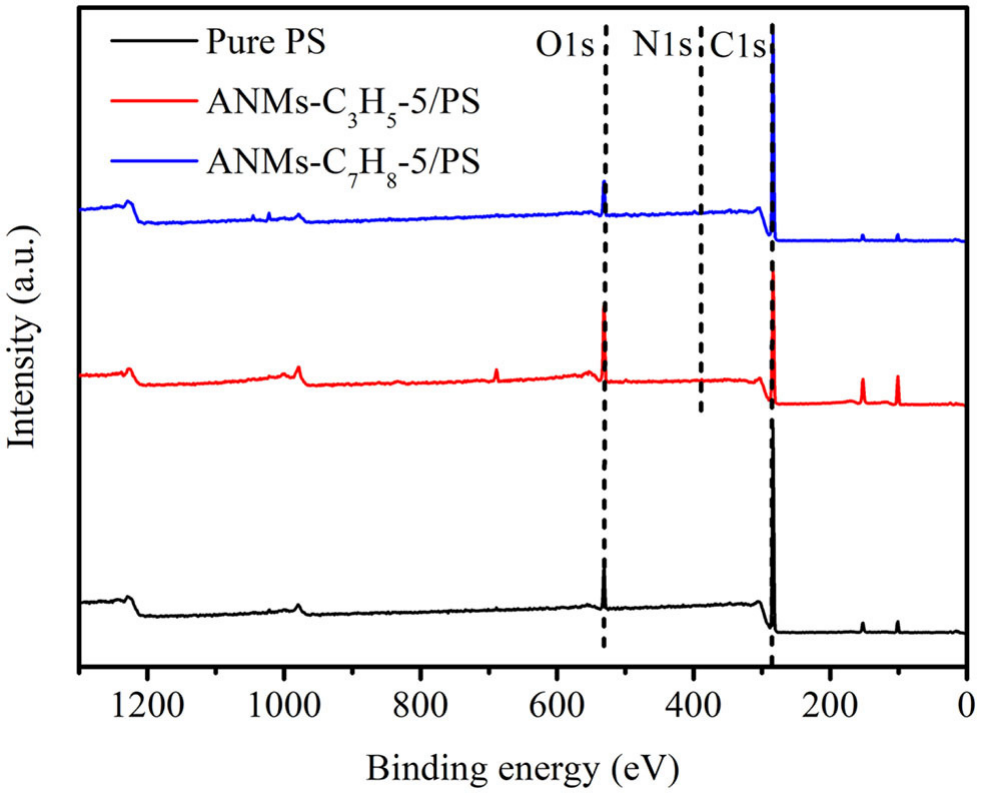
Overlaid XPS spectra of Pure PS, ANMs-C_3_H_5_-5/PS and ANMs-C_7_H_8_-5/PS.

**Figure 6:**
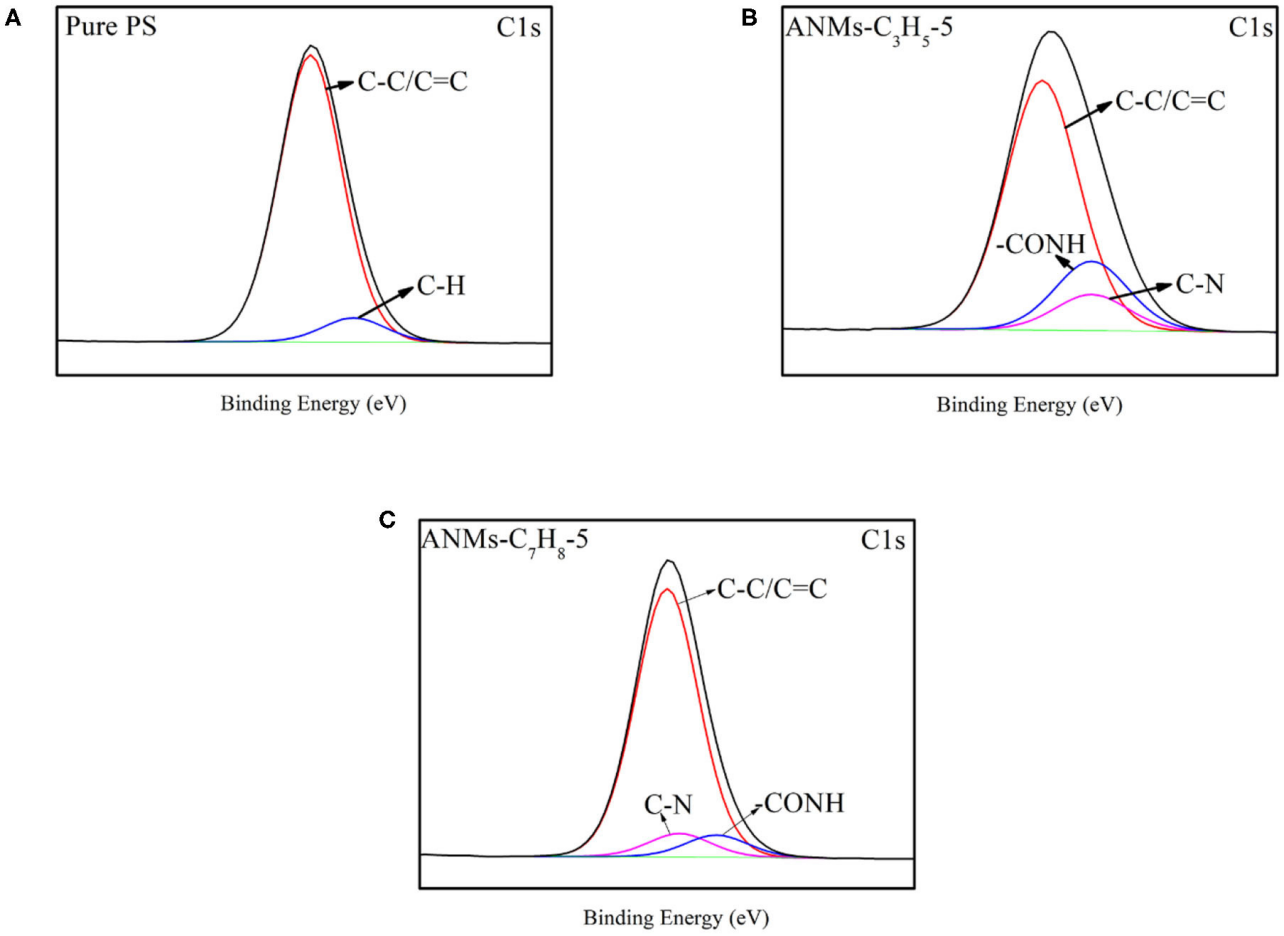
Carbon (1 s) spectra of Pure PS **(A)**, ANMs-C_3_H_5_-5/PS **(B)**, and ANMs-C_7_H_8_-5/PS **(C)**.

The surface chemical compositions of Pure PS and ANMs-C_3_H_5_-5/PS and ANMs-C_7_H_8_-5/PS were determined by XPS as shown in Table 2. There is a significant increase in the oxygen-to-carbon ratio for ANMs-C_3_H_5_-5/PS as compared to pure PS, which may be caused by the introduction of allyl groups on the aramid fiber nanofibers. However, the change in same ratio for ANMs-C_7_H_8_-5/PS was not significant, which might be explained by the similar chemical structure of benzyl to that of PS. Figures 5, 6 show the XPS wide scan spectra of different samples and the C1s spectra of three samples after the split-peak. It can be seen from the C1s spectrogram that after modification by allyl or benzyl, not only the chemical groups of aramid fiber are retained, but also the -C=C bond of allyl and the -C-H bond of benzyl benzene ring are introduced into the aramid fiber (Sun et al., 2012, 2014; Zhang et al., 2017). The different peak intensities of C1s and O1s in the full spectra may also indicate the introduction of allyl or benzyl groups on the aramid nanofibers (Guo et al., 2009; Jia et al., 2010; Cheng et al., 2016).

**Table 2:** Surface elemental compositions and O/C ratios of Pure PS, ANMs-C_3_H_5_-5/PS, and ANMs-C_7_H_8_-5/PS.

**Samples**	**Element mass fraction**			**O/C**
	**N (%)**	**C (%)**	**O (%)**	
Pure PS	0.48	84.27	8.07	0.096
ANMs-C_3_H_5_-5/PS	0.49	64.87	15.92	0.245
ANMs-C_7_H_8_-5/PS	1.08	86.63	7.27	0.084

The above analysis indicates that allyl and benzyl have been successfully introduced into aramid nanofibers, which is consistent with the conclusion of XRD.

#### TEM Analysis

**Figure 7:**
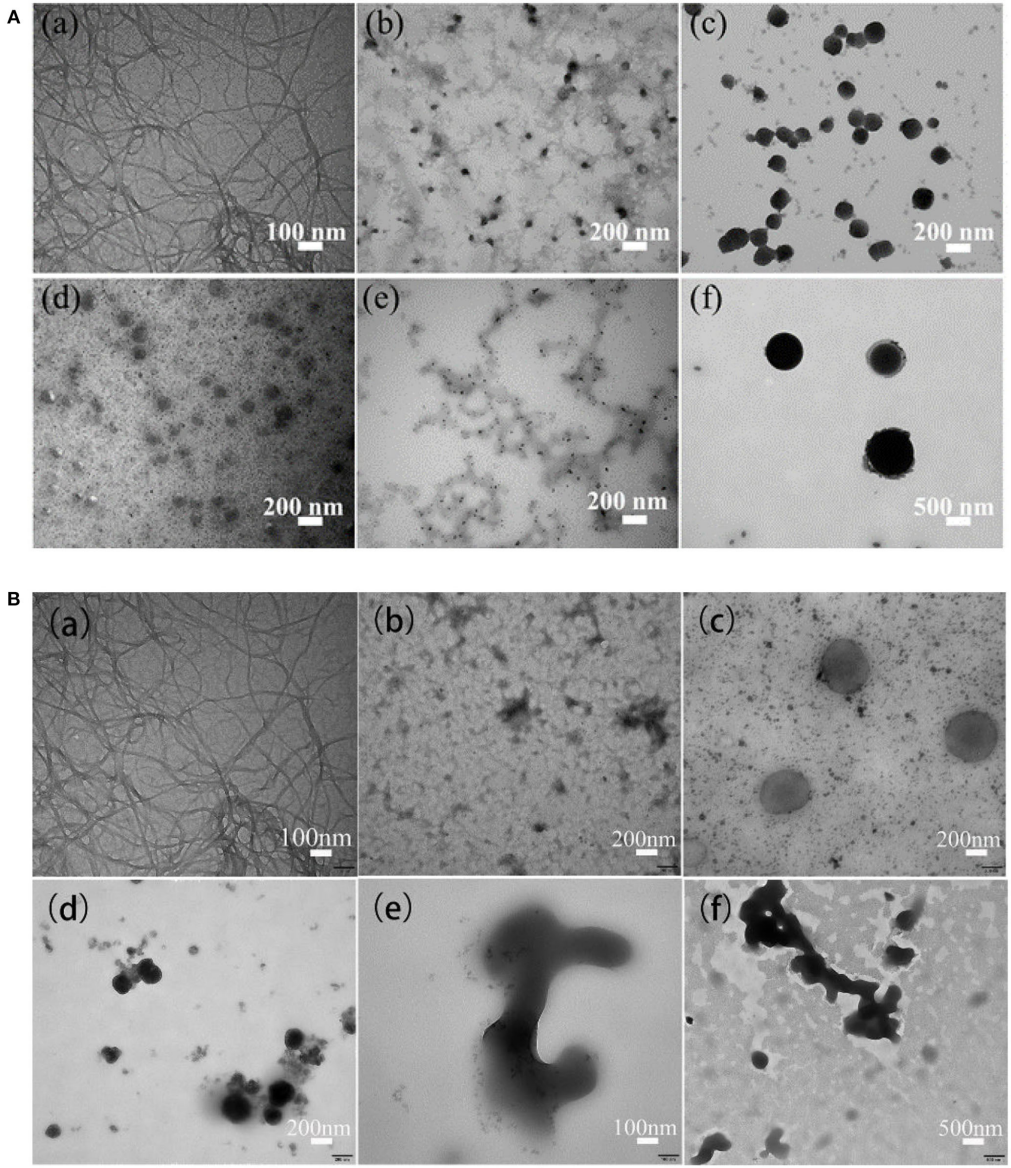
**(A)** TEM images of ANFs and ANMs-C_3_H_5_-X samples: (a) ANFs, (b) ANMs-C_3_H_5_-1(10%), (c) ANMs-C_3_H_5_-2(20%), (d) ANMs-C_3_H_5_-3(50%), (e) ANMs-C_3_H_5_-4(100%), (f) ANMs-C_3_H_5_-5(200%). **(B)** TEM images of ANFs and ANMs-C_7_H_8_-X samples: (a) ANFs, (b) ANMs-C_3_H_5_-1(10%), (c) ANMs-C_3_H_5_-2(20%), (d) ANMs-C_3_H_5_-3(50%), (e) ANMs-C_3_H_5_-4(100%), (f) ANMs-C_3_H_5_-5(200%).

The TEM images in Figure 7 shows the morphologies of ANFs (a) as well as ANMs-C_3_H_5_-X and ANMs-C_7_H_8_-X with increasing degrees of substitution from (b) to (f).

Take benzyl modified ANFs as an example in Figure 7B. The morphology of modified allyl and benzyl samples was compared with that of unmodified aramid nanofibers. In addition to the 10% of the samples with theoretical substitution degree, the structure of nanofibers was retained, but the fiber structure was also decomposed, and the morphology of the ANFs with other degrees of substitution were significantly changed. At 20% substitution (c), there are small spheres as well as large spheres likely formed by aggregation of small spheres. At theory of 50% substitution (d), dense microspheres appear and there is a little overlap and merger between the dense microspheres. At theory of 100% (e) or 200% (f) substitution, the morphology changed greatly and presented irregular shape.

Previous studies have shown that the degree of functionalization affects the morphology of ANFs. Generally, with the improvement of the degree of functionalization of ANFs, the morphology of ANFs will gradually change from nanofibers to other forms, such as nanoflakes and nanospheres. When Cao et al. modified ANFs with phosphoric acid (PA) and glutaraldehyde (GA), it was found that with the improvement of the degree of modification, ANFs gradually transformed into nanosheet form (Cao et al., 2013). Similar morphological changes of ANFs have been observed in the studies of chemically modified multi-wall carbon nanotubes (MWCNTS). When different concentrations of epichlorohydrin were used to functionalize ANFs, with the increase of the degree of modification, ANFs was finally transformed into nano-spherical structure (Pan et al., 2017).

The morphology changes with the degrees of substitution may be explained as follows. The reason for the change of ANFs morphology may be the introduction of allyl or benzyl groups. On the one hand, the hydrogen bond between PPTA molecules weakens and the intermolecular distance increases. On the other hand, the introduced group and PPTA molecular chain are not in the same plane, and the regularity of the molecular structure is destroyed, so that PPTA molecules are not arranged in a neat way, and finally ANFs is disintegrated. The dissociated functionalized PPTA molecules wound together to form a spherical structure. This phenomenon is similar to the mechanism of the formation of spherical nanomaterials after the deprotonation of Heterocyclic aramid in DMSO (Mo et al., 2000). Eventually, with the increase of substitution degree, the number and size of nano-spheres also increase correspondingly.

The structure determines the properties, and when the morphology of modified ANFs changes more than before, it should also have a greater impact on the performance of the composite.

### Fabrication of Composite Films

#### Mechanical Property Test

**Figure 8:**
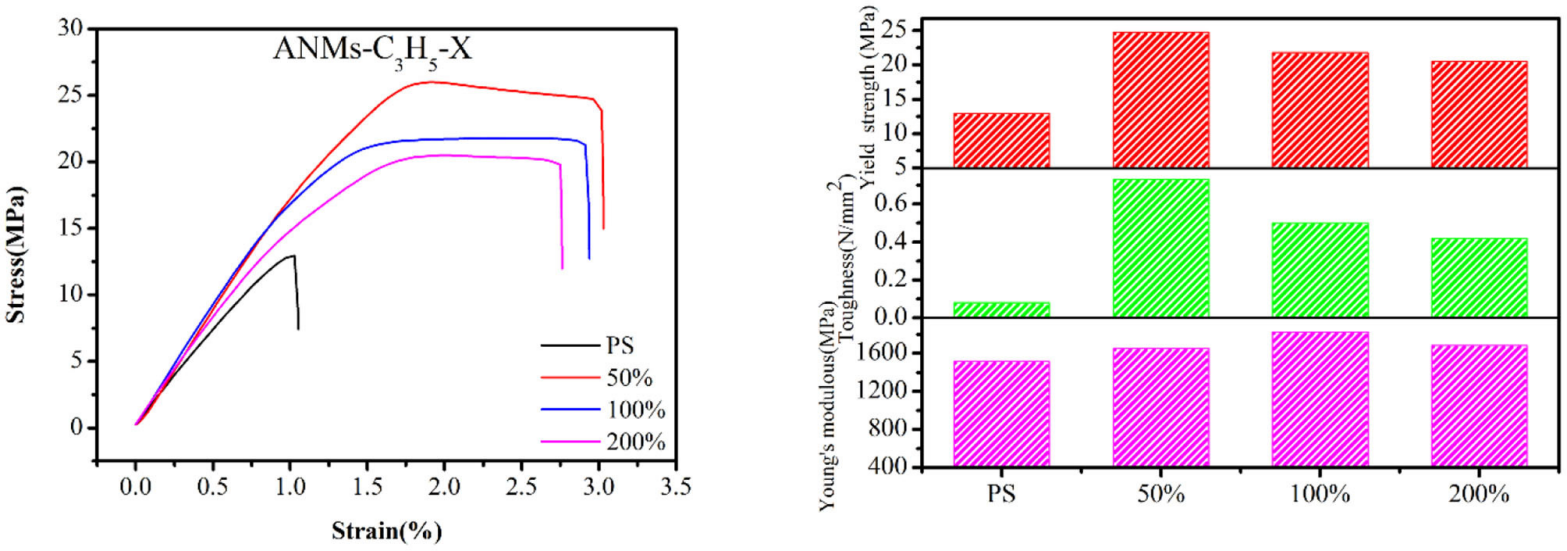
Mechanical testing diagram and results of pure PS and the ANMs-C_3_H_5_-X/PS.

**Figure 9:**
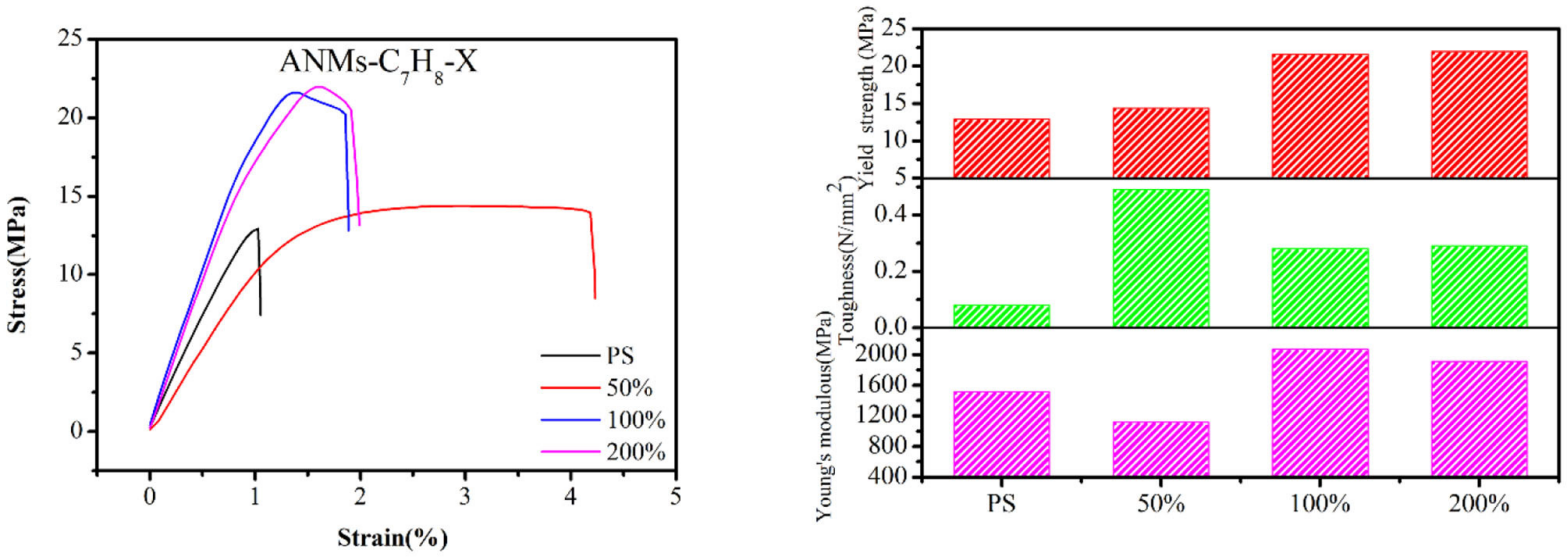
Mechanical testing diagram and results of pure PS and the ANMs-C_7_H_8_-X/PS.

Composite films were prepared by adding R-ANMs with different degrees of substitution as reinforcement to PS at a certain weight ratio. The typical stress-strain curve, tensile strength, Young's modulus and toughness of the films are shown in Figures 8, 9. The values of the Young's modulus, toughness and yield strength are presented in Table 3 with their percentage enhancement rates vs. the pure PS film listed in the parentheses.

**Table 3:** Yield strength, toughness and Young's modulus of pure PS film, ANMs-C_3_H_5_-X/PS, and ANMs-C_7_H_8_-X/PS composite films.

**Sample notation**	**Yield strength (MPa)**	**Toughness (N/mm^**2**^)**	**Young's modulus (MPa)**
Pure PS	12.93	0.08	1,514.00
ANMs-C_3_H_5_-50%	24.78 (91.65%)	0.73 (812.50%)	1,653.66 (9.22%)
ANMs-C_3_H_5_-100%	21.78 (68.45%)	0.50 (525.00%)	1,827.09 (20.68%)
ANMs-C_3_H_5_-200%	20.49 (58.47%)	0.42 (425.00%)	1,685.88 (11.35%)
ANMs-C_7_H_8_-50%	14.39 (11.29%)	0.49 (521.50%)	1,117.97 (−26.16%)
ANMs-C_7_H_8_-100%	21.59 (66.98%)	0.28 (250.00%)	2,069.26 (36.68%)
ANMs-C_7_H_8_-200%	21.97 (69.45%)	0.29 (262.50%)	1,913.88 (26.41%)

The change of ANFs morphology may directly affect the properties of the prepared composite. For example, Cao et al. (2013), discussed in the previous section, prepared a series of thin films in which the mechanical properties of the films increased or decreased when ANFs was transformed from nanofiber to nanoflake structure. Thus, it can be seen when the morphology of ANFs is different, its influence on the mechanical properties of composite materials is different.

For allyl modified ANFs, the stress-strain curve in Figure 8 shows that the yield strength and toughness of the composite PS film improved significantly, while the Young's modulus were enhanced to some extent. The most significant increase in the Young's modulus was at 100% substitution, while yield strength and toughness saw the most significant enhancement at 50% substitution. For benzyl modified ANFs shown in Figure 9, the yield strength increased slightly and the toughness increased greatly at 50% substitution, but the Young's modulus decreased. The enhancement in yield strength and toughness was similar at 100% substitution and 200% substitution, while the Young's modulus at both levels was enhanced more than that in the allyl modified ANFs.

At the same time, the morphology of modified ANFs has a certain effect on the enhancement of PS matrix. From Figure 8, the improvement of mechanical properties of PS by allyl modified ANFs gradually weakens with the increase of substitution degree, but all of them are higher than the mechanical properties of PS original film. For benzyl modified ANFs, in addition to toughness, young's modulus and yield strength were enhanced with the increase of substitution degree compared with the PS film, the modified ANFs improved the mechanical properties of PS.

In sum, the mechanical properties of the PS film, including the yield strength, Young's modulus, and toughness, were enhanced to varying degrees with the addition of allyl and benzyl modified ANFs.

#### TGA Analysis

**Figure 10:**
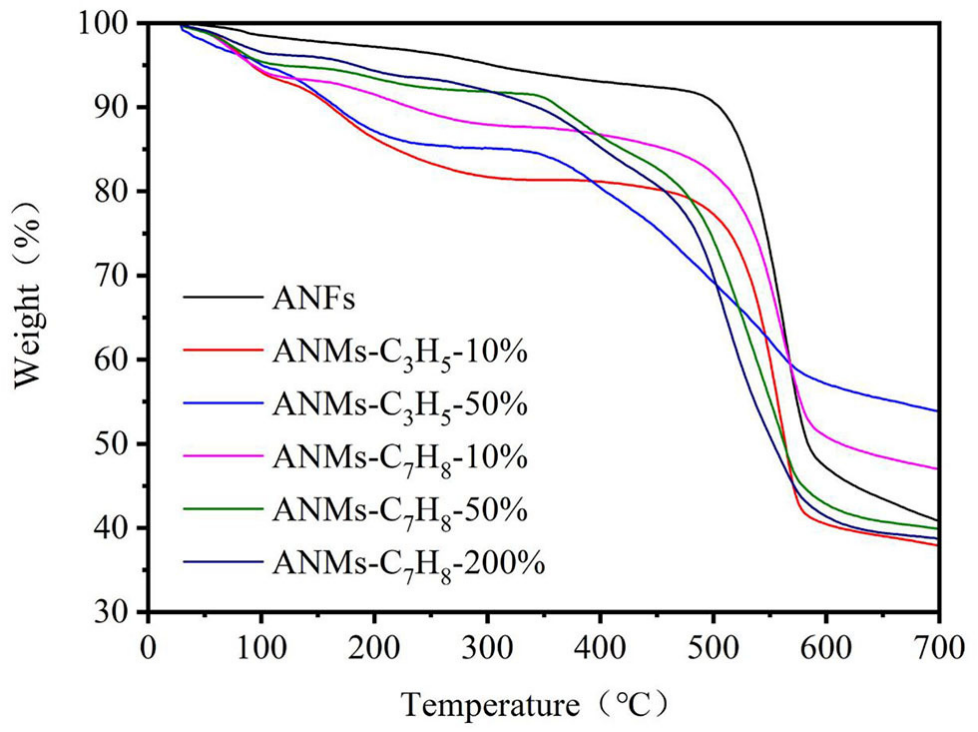
TGA curves of ANFs, ANMs-C_3_H_5_-X, and ANMs-C_7_H_8_-X.

Thermogravimetric analysis (TGA) was used to characterize the thermal stability of ANFs, ANMs-C_3_H_5_-X and ANMs-C_7_H_8_-X nanomaterials. The TGA were presented in Figure 10. From the TGA image, it can be seen that the decomposition process of all samples can be divided into two stages, with a slow quality loss before 500°C and a large quality loss between 500 and 600°C. In the first stage, the quality loss of ANFs after modification was more obvious than that of ANFs. In this stage, the loss may be caused by the decomposition of allyl or benzyl grafted on ANFs which becoming volatile products (Arrieta et al., 2011). In the second stage, ANFs should decompose when there is an obvious mass loss, and the weight ratio of ANFs is about 50% in loss of mass. This result is consistent with similar studies on ANFs in the literature (Fan et al., 2013). On the other hand, the modified ANFs maximum decomposition temperature did not change significantly, indicating that the modification would not affect the thermal stability of ANFs.

## Conclusions

The allyl and benzyl modified aramid nanofibers with different degrees of substitution were successfully fabricated without changing the thermal stability of the aramid nanofibers in this study. Research to a certain quality score (10%) of modified ANFs added to PS as strengthening agent in the substrate material, toughness and young's modulus of the composites has a different level of ascension. This indicates that ANFs modified with allyl or benzyl groups may be an ideal additive for enhancing polymer materials, broadening the use of ANFs as reinforcement in composite materials.

## Data Availability Statement

The original contributions presented in the study are included in the article/supplementary materials, further inquiries can be directed to the corresponding author/s.

## Author Contributions

GP carried out experiments and wrote the manuscript. SC and QR designed experiments, analyzed results, and revised the manuscript. DC and WYa carried out performance-enhancing experiments of the PS films. JC, ZY, and WYi characterized and analyzed experimental results. This work was completed by cooperation of all authors.

## Conflict of Interest

The authors declare that the research was conducted in the absence of any commercial or financial relationships that could be construed as a potential conflict of interest.
